# Image analysis-based quantification of fungal sporulation by automatic conidia counting and gray value correlation

**DOI:** 10.1016/j.mex.2021.101218

**Published:** 2021-01-07

**Authors:** Linda C. Muskat, Yannic Kerkhoff, Pascal Humbert, Tim W. Nattkemper, Jørgen Eilenberg, Anant V. Patel

**Affiliations:** a University of Applied Sciences Bielefeld, Fermentation and Formulation of Biologicals and Chemicals, Faculty of Engineering Sciences and Mathematics, Bielefeld, Germany; b Georg-August-University Goettingen, Agricultural Entomology, Department of Crop Sciences, Goettingen, Germany; c University of Bielefeld, Faculty of Technology, Biodata Mining Group, Germany; d Free University of Berlin, Institute of Chemistry and Biochemistry, Berlin, Germany; e University of Copenhagen, Department of Plant and Environmental Sciences, Insect Pathology and Biological Control, Copenhagen, Denmark

**Keywords:** (Semi-)Automatic conidia counting, Computer-assisted sporulation quantification, Entomopathogenic fungi

## Abstract

The present work describes a new computer-assisted image analysis method for the rapid, simple, objective and reproducible quantification of actively discharged fungal spores which can serve as a manual for laboratories working in this context. The method can be used with conventional laboratory equipment by using bright field microscopes, standard scanners and the open-source software ImageJ. Compared to other conidia quantification methods by computer-assisted image analysis, the presented method bears a higher potential to be applied for large-scale sample quantities. The key to make quantification faster is the calculation of the linear relationship between the gray value and the automatically counted number of conidia that has only to be performed once in the beginning of analysis. Afterwards, the gray value is used as single parameter for quantification. The fast, easy and objective determination of sporulation capacity enables facilitated quality control of fungal formulations designed for biological pest control.

• Rapid, simple, objective and reproducible quantification of fungal sporulation suitable for large-scale sample quantities.
• Requires conventional laboratory equipment and open-source software without technical or computational expertise.
• The number of automatically counted conidia can be correlated with the gray value and after initial calculation of a linear fit, the gray value can be applied as single quantification parameter.

## Graphical abstract

**Figure:**
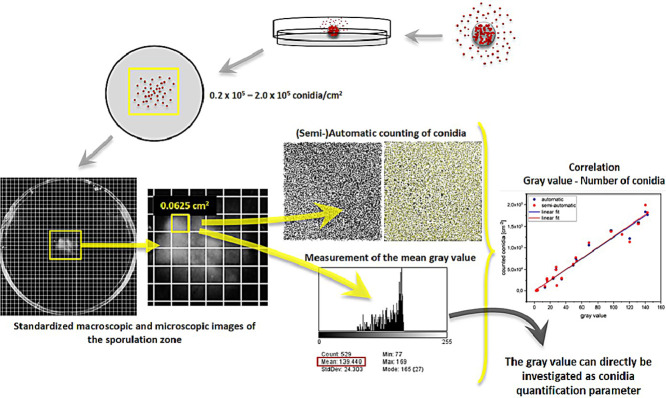

Specifications table

**Table:** 

Subject Area	Agricultural and Biological Sciences
More specific subject area:	Quantification of fungal sporulation
Method name:	Quantification of fungal sporulation by (semi)-automatic counting of conidia and gray value correlation
Name and reference of original method	NA
Resource availability	ImageJ software (https://imagej.net/Fiji/Download; v1.52p)

## 1 Introduction

The formulation of living biocontrol agents as capsules improves their applicability, shelf life and storability and prolongs the sporulation duration after field application by serving as a "microfermenter" formulation [9]. When considering entomopathogenic fungi for use in biological control, one of the most important issues is a reliable method for quantification of virulent conidia formed by encapsulated fungal cells.

Fungi of the order of Entomophthorales bear an exceptional high potential for biological insect pest control because of their narrow host range and fast speed-to-kill [8] and the genus *Pandora* contains several species with potential for biological control. Conidia, the asexual spores which are the infection units of these fungi, are actively discharged and dispersed into the environment. After landing, the conidia of *Pandora* spp. stick, by aid of mucoid substances and a partly detached cell wall, to surfaces like the host insect cuticle [4,7]. Although the role of this mucous layer is not yet completely clear, it makes classical collection and counting of the conidia using common surfactants like polysorbates, e.g. Tween 80, difficult or even impossible.

A standardization of a novel observation method to determine the meaningful parameter of sporulation capacity of *Pandora* spp. is necessary to enable a routine quality control for biocontrol formulations containing living entomophthoralean fungal cells.

Compared to manual counting of discharged conidia, which is a subjective and time-lasting procedure, automatized image analysis is quicker and can improve the comparability of results. Nielsen et al. [6] developed a method for the characterization of conidial size and shape from microscopic images of the conidia of different *Pandora* species. Korsnes et al. [5] accelerated counting of conidia from spore trap samples by automatized detection and identification of conidia of *Pandora neoaphidis*. Bonner et al. [1] established a semi-automatized method for the identification and counting of conidia of *P. neoaphidis* by selection of conidia based on the gray scale for shape recognition. These publications demonstrate that automatized detection and counting of fungal spores is a subject of relevance, with regards to biological control and also, it is relevant for understanding the biology of entomophthoralean fungi as well as other fungi. These published methods make counting faster, easier and more objective, but so far they have solely been proven useful for small sample quantities and from microscopic images.

The aim of the present study was the development of a rapid, simple and objective method for the quantification of discharged conidia of the encapsulated entomophthoralean fungus *Pandora* sp. nov. (ARSEF 13372), which has potential for psyllid control. Our starting assumption and hypothesis: We assumed that conidia that were dispersed onto a smooth surface would reflect diffuse light and we suggested that this emission would correlate with the number of discharged conidia. By calculating the correlation of the gray value as magnitude of the reflected light with the automatically counted number of conidia, we aimed to develop a method to accelerate the speed of conidia quantification.

## 2 Method details

### 2.1 Fungal strain

The fungus *Pandora* sp. nov. (ARSEF 13372) used in this work was isolated from an infected pear psyllid (*Cacopsylla pyri*) collected from a Danish pear orchard in 2016 by the Eilenberg-group (Department of Plant and Environmental Sciences, University of Copenhagen). The fungus is a novel, not yet described species of the entomophthoralean genera *Pandora* [3].

### 2.2 Cultivation of the entomopathogenic fungus Pandora sp. (ARSEF13372)

The fungus was grown as described by Hajek et al. [2] on Saboraud Dextrose Agar (SDA) supplemented with 20% of a mixture of egg yolk (60%) and fresh skimmed milk (40%) (SDAME) on Petri dishes (diameter 90 mm) sealed with parafilm® and incubated at 18 °C in the dark. To maintain the culture, mycelia plugs (0.5 cm^2^) were cut and transferred to fresh media, when the mycelium reached the boarder of the plate. The fungus used for experiments was transferred less than 3 times.

### 2.3 Production of hyphal material of *Pandora* sp. (ARSEF 13372) in liquid culture

The cultivation method was adapted from Shah et al. [11]. First, in order to transfer the fungus from the solid media into liquid culture, three pieces of mycelia (0.5 cm^2^) grown on SDAME agar plates were cut with a scalpel and transferred to 100 ml of a pre-culture medium composed of 10% skimmed milk in ultra-pure water (MilliQ) in 250 ml shaking flasks with 4 baffles and incubated at 18 °C and 180 rpm with an amplitude of 20 mm in the dark for 48 h. The main-culture, composed of dextrose (1.6%, w/v), yeast extract (1%, w/v), sodium chloride (NaCl; 0.9%, w/v) and skimmed-milk powder (10%, w/v) was inoculated with 10% (v/v) of the pre-culture and incubated at the same conditions for 72 h.

### 2.4 Encapsulation of the fungus within calcium alginate beads

The hyphal material from the liquid shaking culture was separated from the medium by centrifugation (4500 rpm; 10 min; 18 °C) and washed twice in NaCl solution (0.9%) before encapsulation within calcium alginate beads. The formulation composition was adapted from Shah et al. [11] with some modifications. The formulation solution was composed of sodium alginate (1.5%, w/w), maize starch (10%, w/w), NaCl (0.9%, w/w) and hyphal material of *Pandora* sp. nov. (ARSEF 13372) (10%, w/w). After mixing the components in a beaker by magnetic stirring at 250 rpm, the mixture was dripped by means of a 20 ml syringe (Braun, Germany) equipped with a needle (0.90 × 40 mm; Braun, Germany) from a height of 10 cm into a stirred (250 rpm) calcium chloride solution (0.1 M). The formed beads were gelled for 20 min in the cross-linking solution and afterwards washed with a NaCl solution (0.9%) for 30 s. All encapsulation steps were carried out at room temperature (22–24 °C). The beads size was 4.44 mm (±0.24) diameter and the initial water activity (a_W_) was about 0.955.

### 2.5 Conidia collection

Freshly prepared calcium alginate beads containing *Pandora* sp. nov. (ARSEF 13372) were transferred to Petri dishes, filled with 20 ml water agar (2%, w/v). The distance between the agar and the lid of the Petri dish on which the conidia were collected in this experiment was 7 mm.

**Fig. 1:**
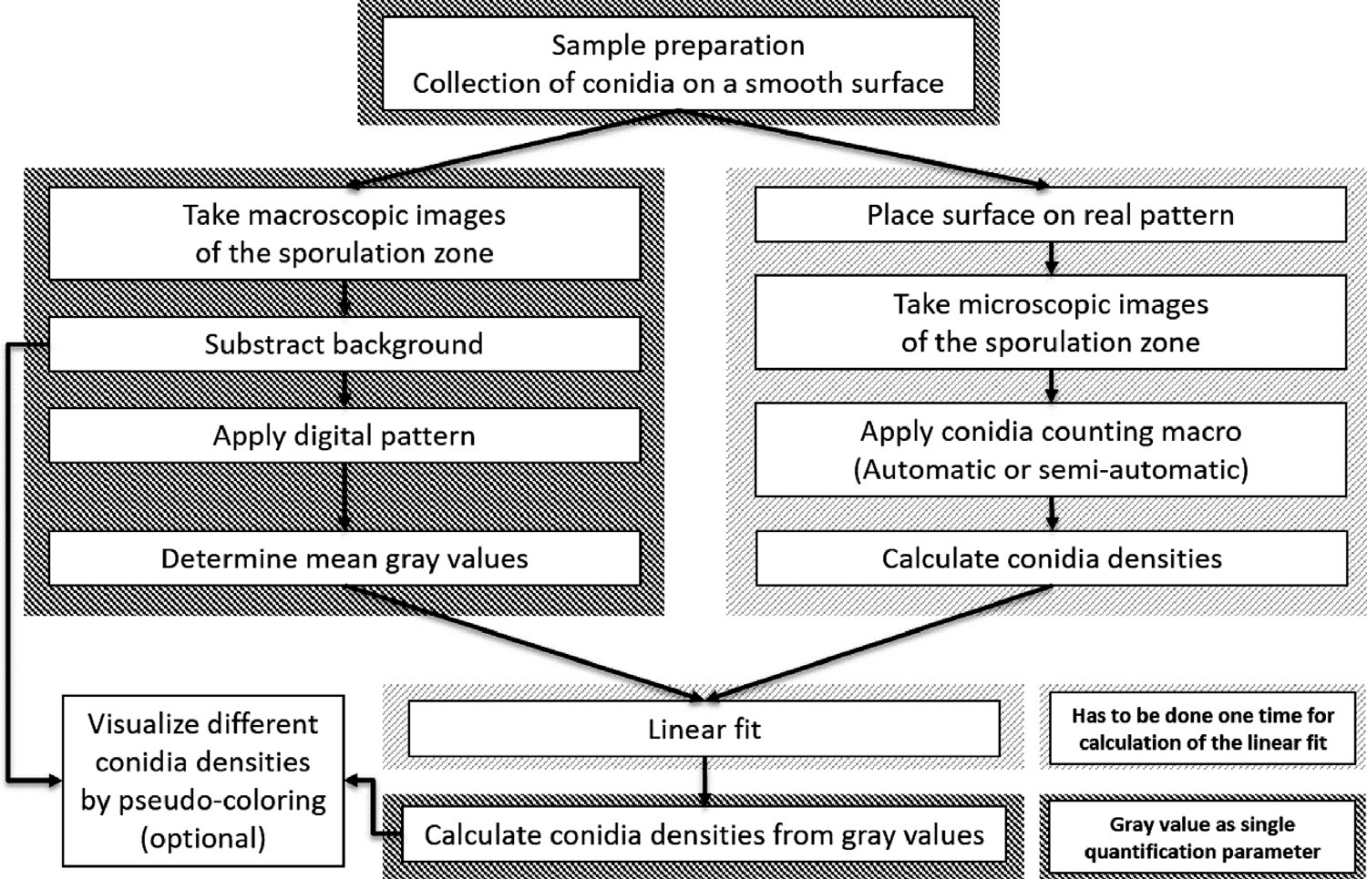
Workflow of the sporulation quantification. The sample must be prepared by collecting conidia on a smooth surface. The surface with the sporulation zone has to be placed on a grid pattern and microscope images have to be taken. One of the provided macros must be applied to get the conidia densities and fit them against the mean gray values of the corresponding digital grid from macroscopic images. The fit allows to calculate conidia densities from the gray values and allows optional pseudo-coloring for visualization purposes.

The Petri dishes were sealed with Parafilm® and incubated at 18 °C in the dark. After 72 h, the conidia collected on the lids of the Petri dishes were used as sample within the next experimental steps for sporulation quantification, which are illustrated in Fig. 1.

### 2.6 Sample preparation

The method is suitable for fungi or other organisms that disperse their conidia or any other actively discharged units into the environment. The discharged units must be identifiable by bright field microscopy.

A Petri dish lid, a glass slide or any other kind of smooth surface used for the collection of particles must be clear, clean and dry. We suggest cleaning a surface with an alcoholic solvent and a lint-free paper tissue, but gently to avoid creating scratches. The blanks should be prepared the same way. If the sample collection surface is wet after the collection of conidia, e.g. under high humidity conditions like required for the sporulation of the fungus used in this work, it should be dried prior to the image generation.

### 2.7 Macroscopic image generation of sporulation zone

**Fig. 2:**
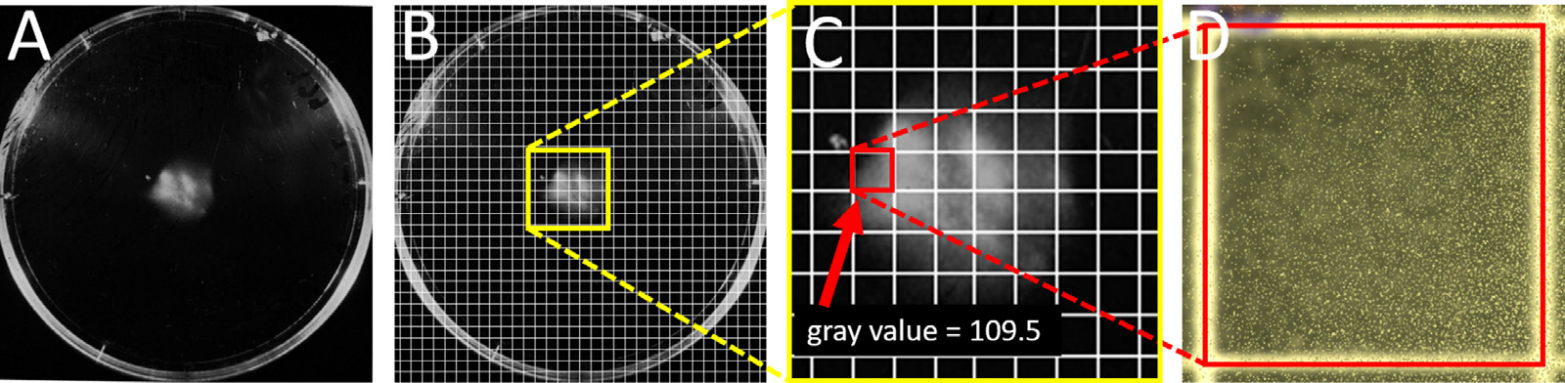
Generation of gray value and corresponding microscopic sporulation image. A) Macroscopic image of the conidia collected on a Petri dish lid after background subtraction. B) Background corrected images overlaid with a digital grid for separation of the sporulation zone into disjunct areas. C) Gray value measurement of a selected grid cell. D) Microscopic image of the selected grid cell for conidia counting.

The surface for particle collection (e.g. Petri dish lid) is placed in a macroscopic camera system, e.g. scanning or gel reading apparatus. The sporulation zone is checked for disturbing scratches and the parameters are adjusted so that a clear picture of the sporulation zone can be recorded. (Fig. 2, A). In addition, an image of the empty camera system is taken using the same parameters as with the surface as blank. All images should be saved as 8-bit images (e.g. tif format).

### 2.8 Image processing software ImageJ

To perform the following image processing steps, the open-source software ImageJ (v1.52p, Fiji) is required. We recommend the download of Fiji, which is a version of ImageJ with some useful pre-installed plug-ins. Further information on software download, plug-in options and theoretical background are available on the ImageJ homepage^1^^,^^2^.

### 2.9 Macroscopic image correction and determination of the mean gray values

Empty background image subtraction is carried out using the function Image Calculator (Process>Image Calculator…) from the open-source software ImageJ (v1.52p, Fiji) so that the image of the empty gel reading apparatus (blank) is subtracted from the images taken with a surface. The background corrected images are used to overlay a digital grid (Analyze>Tools>Grid) (Fig. 2, B). This way of separation of the sporulation zone into disjunct areas is achieved (Fig. 2, C). It should be noted that in “Set Measurements” (Analyze>Set Measurements…) the “Mean gray value” checkbox must be activated when repeating the experiment. Finally, the areas are marked (“Rectangle”) and the gray values can be read out (Ctrl+M). For each area, the average gray value *g* has to be stored.

### 2.10 Microscopic image generation of the sporulation zone

The surface on which the particles are collected must be placed on a grid pattern (which corresponds to the digital pattern) and both are inspected with a bright field microscope. Certain areas of the sporulation zone are selected and an image (8-bit format) will be recorded. Afterwards, the resulting image areas will be identified on the macroscopic images from the scanning or gel reading apparatus (Fig. 2, C & D). In our set-up, grids of 2.5 × 2.5 mm were used. Only grids containing particles will be considered for further analysis.

### 2.11 Automatic and semi-automatic counting of conidia

In the following, the process of automatic conidia counting (ACC) is described. Afterwards, the semi-automatic conidia counting (SACC) method ACC will be described, which is a minor variation of the ACC. Both methods use the Rolling Ball Background Subtraction function from Michael Castle and Janice Keller (Mental Health Research Institute, University of Michigan) which is based on the concept of the rolling ball algorithm described by Sternberg [12]. The execution of both methods is shown in the supplementary video (Sup. 1).

First, all of the microscopic images of the sporulation zone areas are investigated manually and the inside of the grid cells are cropped (“Rectangle” & “Duplicate”) and saved as a stack of image patches. Next, one of the provided ImageJ macros will be applied to the stack. To this end, the macros have to be provided as .txt file and executed via the standard ImageJ functionality (Plugins>Macros). Please note that the “Adjustable Watershed” algorithm has to be installed, i.e. the Adjustable_Watershed.class^3^ from Michael Schmid has to be in the ImageJ folder “Plug-ins”. The result of this process is a stack of binary image patches with segmented spores, corresponding to the input image stack.

In all patches of the binary stack, the number of particles (white blobs) is determined with the Particle Analyzer (Analyze>Analyze Particles) of ImageJ. The check-box “Summarize” has to be activated to get the number of particles on each image and other parameters like size and shape that can be analyzed, if the corresponding check-boxes at “Set Measurement” are activated.

To perform the semi-automatic conidia counting (SACC), the same macro is applied as in the ACC before with the distinction that the intensity threshold *t* of the images can be manually adjusted. Pixels with an intensity value below the threshold *t* are regarded as background and shown in blue. Pixels with an intensity value above the threshold are regarded as conidia and keep their original intensity values. The intensity threshold *t* has to be adjusted until spores and background are best separated. Afterwards, by call Process>Find Maxima… and selection of “Above lower threshold” and “Preview point selection” the local intensity maxima will be shown which should correspond to the conidia. By variation of the option “Prominence” in the “Find Maxima” box the number of detected maxima (conidia) in the result can be adjusted. Lower “Prominence” will result in more maxima, higher “Prominence” will result in less maxima. Now, a suitable “Prominence” value has to be found for a noticeable number of particles or by selection of “Segmented Particles” at “Output type:” to get a segmented binary image like in the automatic macro. The segmented binary image can then be analyzed by the Particle Analyzer.

### 2.12 Calculation of a linear fit between automatically counted conidia and gray value

To analyze the resulting data for correlation, first, all of the conidia densities are computed from the number of conidia counted within all of the specific areas. Next, the gray values (x-axis) are plotted against the corresponding conidia densities *C* (y-axis) and a linear fit will be carried out with some arbitrary basic data analysis software like R or any other suitable. The result formula provides the final function to estimate the conidia density represented by a specific observed gray value *g*.

### 2.13 Visualization of different conidia densities by pseudo-coloring

For visualization, the macroscopic 8-bit images from the scanning or gel reading apparatus are opened and the ImageJ function Image>Lookup Tables>6 shades is applied. The image of the sporulation zone will be pseudo-colored to represent areas with different conidia densities (see Fig. 6 for an example).

## 3 Method validation

**Fig. 3:**
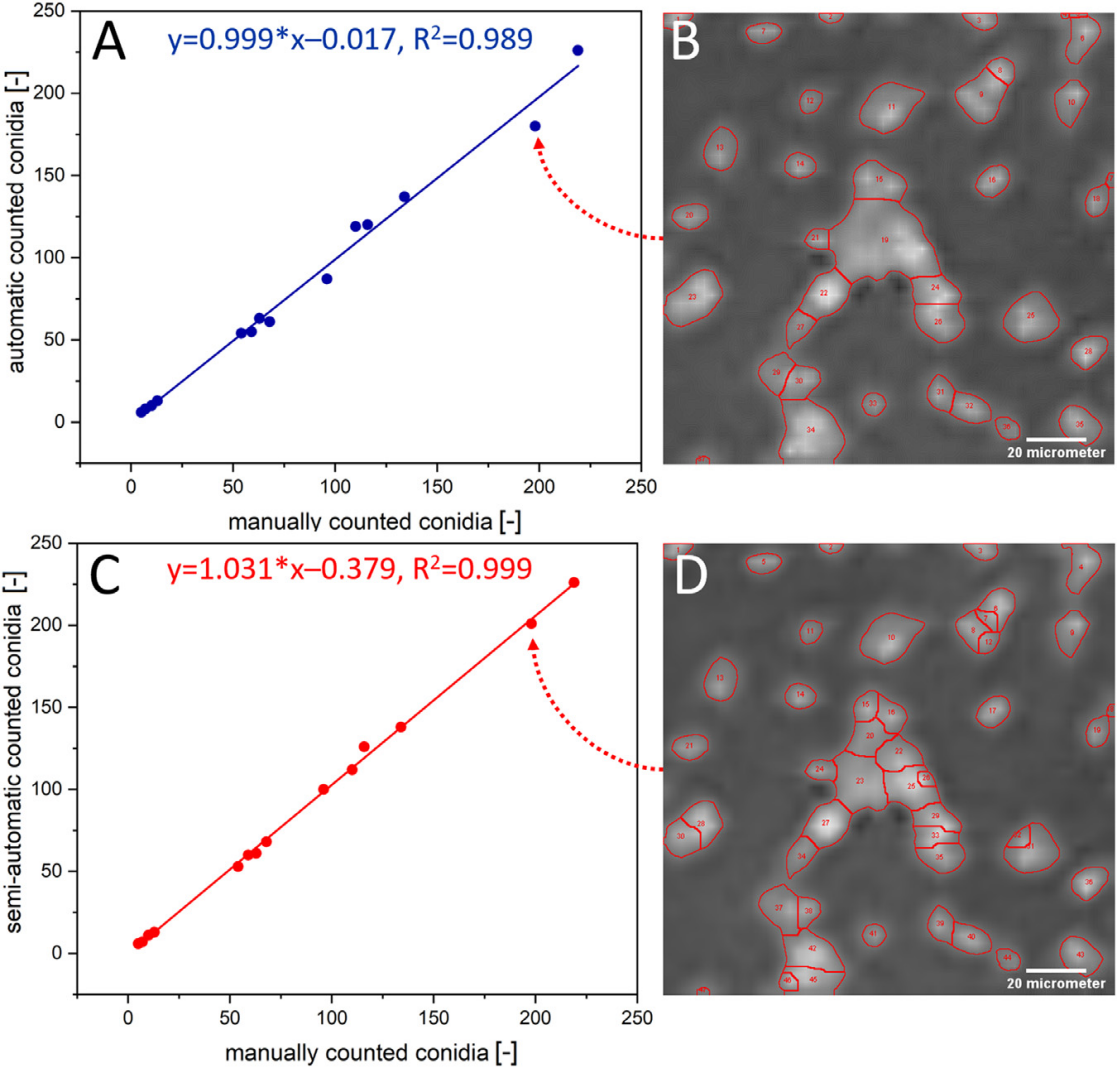
Correlation between (semi-)automatically and manually counted conidia. A) Plot of the automatically and manually counted conidia including linear fit with slope of 0.999 and R^2^=0.989. B). Visualization of the automatic performed segmentation and counting on an example image crop (scale 20 µm) containing single conidia and clusters. The big cluster shows low degree of segmentation (underestimation) and thus leads to a higher deviation from the manually counted conidia number (red arrow). C) Plot of the semi-automatically and manually counted conidia including a linear fit with slope of 1.031 and R^2^=0.999. D) Visualization of the semi-automatically performed segmentation and counting on an example image crop (scale 20 µm) containing single conidia and clusters. Here, the big clusters were further segmented after manual inspection for more reliable results (red arrow).

Two image analysis workflows as previously described were developed in order to automatize the counting of conidia from bright field microscopic images. To determine the accuracy and precision of both workflows, 14 image crops of 500 × 500 µm^2^ containing different numbers of conidia were counted manually and (semi)-automatic. The results were plotted and a linear fit was applied, as a linear correlation was expected (Fig. 3).

As expected, both workflows showed a high correlation (*R^2^* = 0.989 and 0.999) and therefore high accuracy and a high precision (automatic workflow underestimated the conidia number by 1%; semi-automatic workflow overestimated by 3%). Both workflows were therefore considered to be as reliable as manual counting but at the same time considerably faster. The semi-automatic workflow is a bit more time-consuming (approximately 1 min per image) than the fully automatic workflow (due to manual setting of parameters), however it is more precise in detecting conidia in clusters as seen by comparing the middle of Fig. 3B and D.

**Fig. 4:**
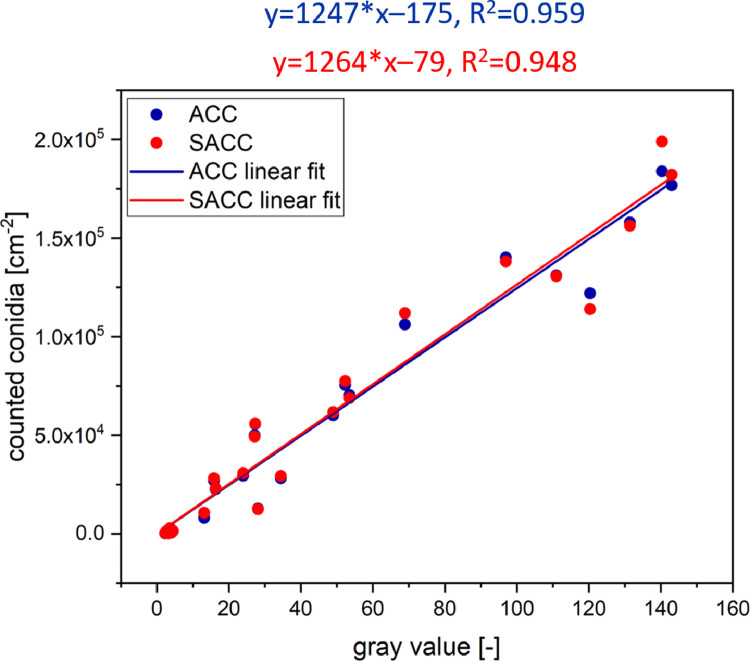
Correlation of the (semi-)automatically counted conidia on microscope images and gray values of the corresponding areas of the sporulation zone. The linear fit of the automatic workflow has a slope of 1247, a y_0_ of -175 and an R^2^ of 0.959. The linear fit of the semi-automatic workflow has a slope of 1264, a y_0_ of -79 and an R^2^ of 0.948. The error of the slope for both fits is approximately ±60 and the error of the y_0_ for both fits is approximately ±3650.

Both workflows were then applied to a data set of 25 microscopy bright field images of 2.5 × 2.5 mm^2^ containing different amounts of conidia in order to determine the conidia density (number of conidia per cm^2^). The counted conidia densities were then plotted against the gray values (8 bit, 0–255) of the corresponding area of the sporulation zone to determine the correlation between conidia density and resulting gray value. A linear fit was applied as a linear correlation was expected (Fig. 4).

While with both workflows the conidia densities determined showed little deviation, the linear fit was mostly identical for both (slope = 1247 and 1264). The correlation (*R^2^* = 0.959 and 0.948) and therefore the precision was very high and proves a high correlation between the conidia density and the gray value of the sporulation zone. This indicates that it is possible to determine the conidia density by analyzing the gray values of the sporulation zone respectively.

**Fig. 5:**
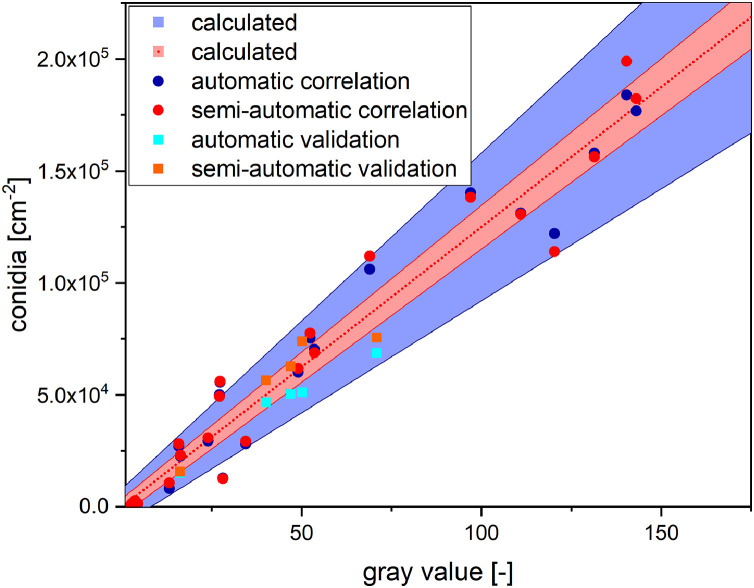
Comparison of conidia densities calculated from gray values and (semi-)automatic counted conidia densities. Blue and red dots represent the conidia densities counted in the correlation data set. Cyan and orange squares represent the conidia densities counted in the validation date set. The red area represents the theoretical conidia densities calculated from gray values by Eq. 1. The blue area represents the theoretical conidia densities calculated from gray values by Eq. 2.

To proof this, a third data set of 5 bright field microscope images of 2.5 × 2.5 mm^2^ containing different amount of conidia was analyzed for validation. The theoretical conidia density *C* of the gray values *g* from 1 to 175 was calculated using the fits of Fig. 4 simplified to:

(1) $$C\left[cm^{-2}\right]=1250\pm 60\cdot g-100\pm 3650$$

and plotted against the corresponding gray values, resulting in a prediction range for conidia density. The conidia of the correlation and validation image sets were counted by the automatic and semi-automatic workflow and plotted (Fig. 5) to compare the calculated and the actual conidia densities. The counted conidia from the previous correlation data set were also plotted.

Fig. 5 shows that the counted conidia densities of the validation data set have a similar distribution like that of the correlation data set. All counted conidia densities increase in linear manner with the gray values of the corresponding areas in the sporulation zone. Fig. 5 also shows that the prediction model of Eq. 1 only includes approximately 50% of the counted conidia densities. The other half of the counted conidia densities are outside auf the error interval. To tackle this issue, the error interval of the model of Eq. 1 was expanded:

(2) $$C\left[cm^{-2}\right]=1250\pm 250\cdot \;g\pm 8000$$

The error interval of the model of Eq. 2 is now broader and this includes approximately 90% of the counted conidia densities of the correlation data set and 100% of the counted conidia densities of the validation data set. The new model is therefore less precise but has a higher accuracy. It can therefore be assumed that if Eq. 2 is used to calculate the conidia densities from gray values, the true value lies within the calculated range in 90% of cases.

Eq. 2 was therefore used to determine the theoretical conidia densities of two additional sporulation zones (Fig. 4) to allow a quantitative comparison.

**Fig. 6:**
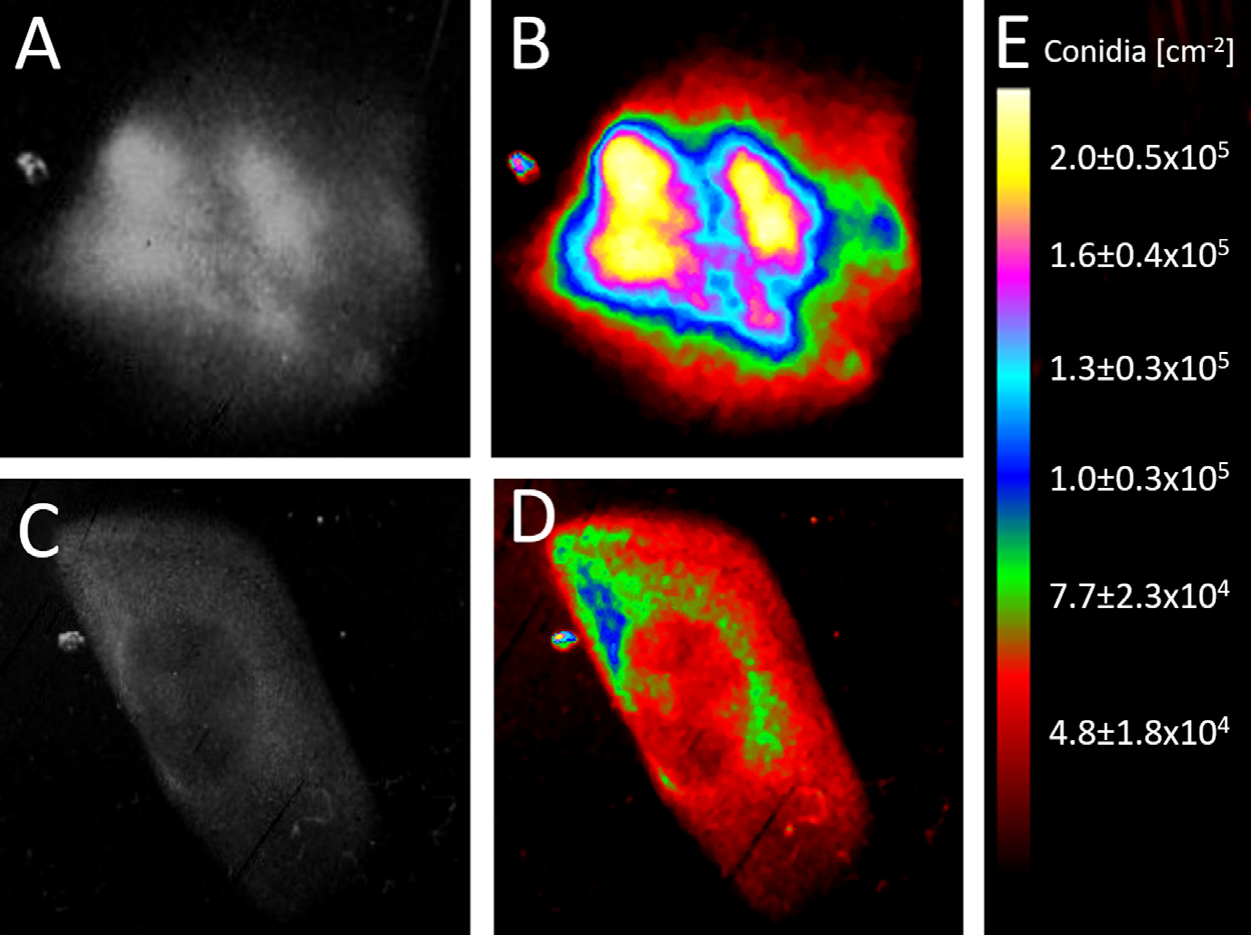
Gray value image and calculated conidia densities of sporulation zones. A) 8-bit gray value image of the first sporulation zone. B) 6 shades pseudo-coloring of the first sporulation zone. C) 8-bit gray value image of the second sporulation zone. D) 6 shades pseudo-coloring of the second sporulation zone. E) Color scale of the pseudo-coloring with corresponding conidia densities C[cm^−2^] calculated from gray values g by Eq. 2.

The 8-bit images were pseudo-colored by the 6 shades look-up-table make the zones with different gray values and thus different conidia densities respectively more distinguishable for the observer. It shows that the outer area of the first sporulation zone (Fig. 6A) contains about 5 × 10^4^ conidia per cm^2^ and two areas with approximately 4-fold higher conidia densities of about 1.9 × 10^5^ conidia per cm^2^ (Fig. 6B). The overall conidia density of the second sporulation zone (Fig. 6C) is significantly lower than that of the first. It contains a small area (blue) with a 2-fold higher conidia density (1 × 10^5^) (Fig. 6D) than the outer area of the zone (approximately 5 × 10^4^) which is just half the density of the yellow zones of Fig. 6B.

## 4 Limits of the method and discussion

**Fig. 7:**
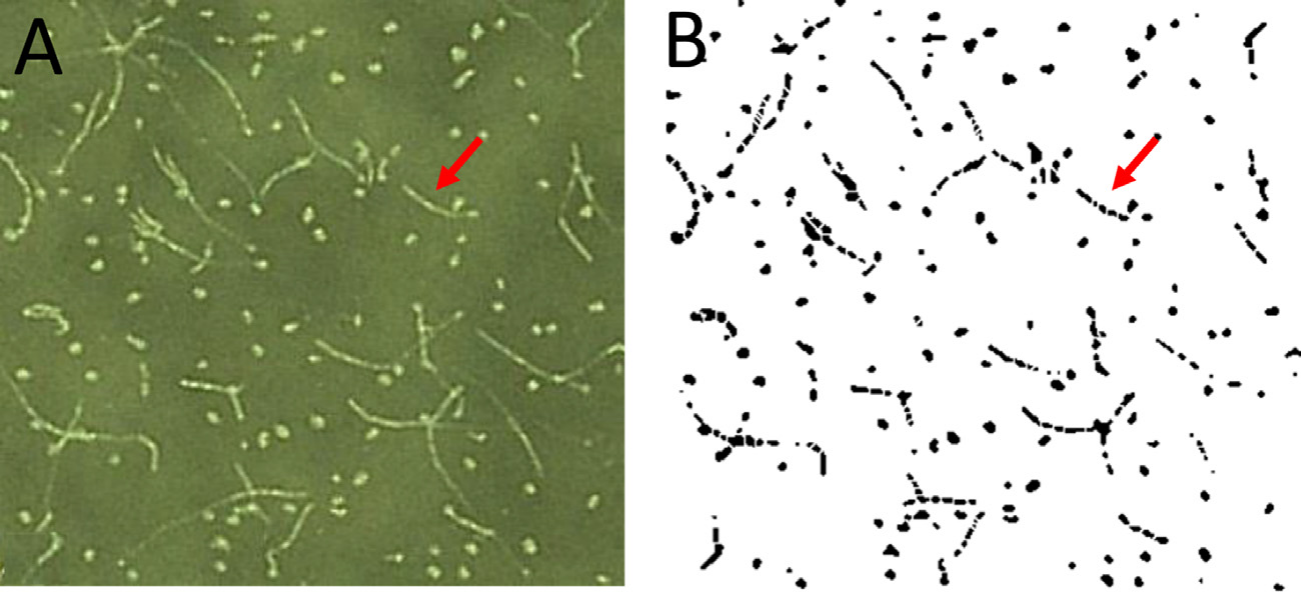
The germ-tubes of the conidia are separated by the watershed algorithm and counted as single particles.

Working with living fungal cells bears some error sources that can negatively affect the sporulation quantification by the developed method. In the present work, the conidia density on the Petri dish lid was insufficiently low to be detected by the developed method within the first 48 hours of sporulation from capsules. As far as the detectable conidia number threshold is not reached, the automatized counting can be applied but not the quantification by the gray value. Moreover, there is an upper limit of the conidia number quantifiable by the developed method based on a maximum gray value threshold. Furthermore, at high conidia densities, conidia are tending to stick together. The problem of segmenting sticking conidia was solved by the application of the watershed algorithm (see **(Semi-)Automatic counting of conidia** in the methodology). Another error source, also existing for other fungi, is the germination of conidia under suitable conditions. As shown in Fig. 7, after application of the watershed algorithm, the germ-tubes (Fig. 7A) are separated and counted as single particles (Fig. 7B).

**Fig. 8:**
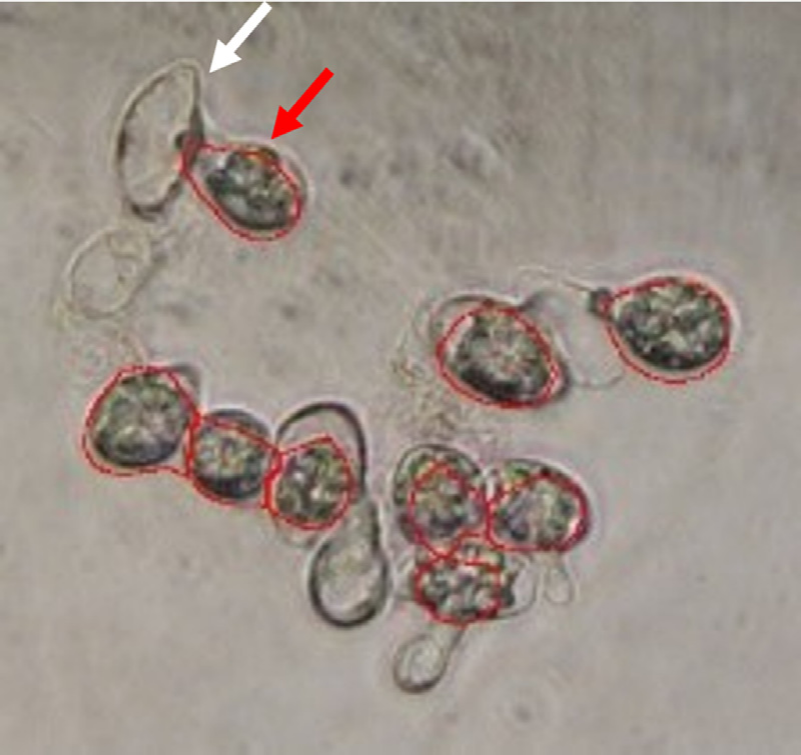
Primary conidia forming secondary conidia (red arrow) and leaving remnants (white arrow). Due to low intensity, the remnants are ignored and only the secondary conidia are counted (red outlines).

In the special case of the Entomophthorales, another point worth noting is the formation of secondary conidia when landing on a surface not suitable for germination. Each primary conidium can form just one secondary conidium, so a secondary conidium represents a primary conidium having landed. The leaving remnant of the primary conidium can, however, cause a potentially increase in the gray value signal. In the case of *Pandora* spp., the error sources of germination and higher order conidia could be eliminated by a shorter sample collection time about 4 hours during high sporulation rate intervals to avoid germination into secondary conidia (Eilenberg, personal observations). In case of a few secondary conidia present, the strong difference in intensity of the secondary conidia and the remnants from primary conidia can be clearly distinguished and it is unlikely that remnants will interfere with the analysis (Fig. 8). Nevertheless, the influence of remnants on the gray value should be evaluated.

## 5 Conclusion

Compared to manual counting of discharged conidia which is a subjective and time-wasting procedure, automatized image analysis improves the comparability of results. Existing computer-assisted methods for conidia quantification are generally not well suited for large-scale sample quantities like it is required for the development of biopesticides based on fungi actively discharging their infective conidia into the environment. The aim of this study was the development of a method for faster and more precise high-throughput quantification of fungal sporulation by computer-assisted image analysis. It was demonstrated that the correlation between the gray value of the sporulation zone, as magnitude of the reflected light by conidia and the actual number of automatically counted conidia can be applied for a comparable quantification of conidial discharge. To give consideration to conidia counting, automatic and semi-automatic image analysis were tested for their practicability. Automatic counting has proved as less subjective and easier for the experimenter, but semi-automatic counting shows higher correlation with the actual number of conidia. To eliminate one of the main error sources of sticking conidia packages, the watershed algorithm was applied for conidia separation. The linear relationship between the number of conidia and the gray value can be investigated for calculation within the range of 0.2 × 10^5^ to 2.0 × 10^5^ conidia/cm^2^. The statistical power of the developed quantification method was validated by comparing the automatized counting results with manual counting to demonstrate the applicability of the developed method. A modified protocol also provides the opportunity to make conidia densities more visible and comparable by more obvious pseudo-coloring. The present method can be performed with low cost, conventional laboratory equipment and the open-source software ImageJ that enables the adaptability of the method by other scientists. Furthermore, the developed method could be combined with established methods for shape and pattern recognition, e.g. those presented by Nielsen et al. [6] or Ranzato et al. [10] and might be adapted for other fungi or microscopic particles as well. It will also prove useful to quantify spores produced in solid state fermentation on technical scale and provide a reliable option for quality control of these bioprocesses. This study demonstrates the high potential of the connection between classical biological techniques and automatized image analysis and should be confirmed by further publications like the present one to make these methods usable in the scientific community.

## CRediT authorship contribution statement

**Linda C. Muskat:** Conceptualization, Methodology, Validation, Formal analysis, Investigation, Resources, Writing - original draft, Visualization. **Yannic Kerkhoff:** Methodology, Software, Validation, Formal analysis, Investigation, Data curation, Writing - original draft, Visualization. **Pascal Humbert:** Project administration, Writing - review & editing. **Tim W. Nattkemper:** Supervision, Writing - review & editing. **Jørgen Eilenberg:** Resources, Supervision, Writing - review & editing. **Anant V. Patel:** Supervision, Writing - review & editing, Funding acquisition.

## Declaration of Competing Interest

The authors declare that they have no known competing financial interests or personal relationships that could have appeared to influence the work reported in this paper.

## Appendix Supplementary materials

Image, video 1

## Footnotes

1 https://imagej.nih.gov/ij/index.html.

2 https://imagej.net/Welcome.

3 https://imagejdocu.tudor.lu/plugin/segmentation/adjustable_watershed/start.
